# Successful treatment with fenestration followed by daily decortication and negative-pressure wound therapy for acute exacerbation of chronic empyema: a case report

**DOI:** 10.1186/s44215-024-00151-9

**Published:** 2024-05-27

**Authors:** Junichi Morimoto, Taiki Fujiwara, Ryo Karita, Jotaro Yusa, Mitsutoshi Shiba, Tomohiko Iida

**Affiliations:** Department of General Thoracic Surgery, Kimitsu Chuo Hospital, 1010 Sakurai, Kisarazu, Chiba 292-8535 Japan

**Keywords:** Chronic empyema, Decortication, Negative pressure wound therapy

## Abstract

**Background:**

Most cases of chronic empyema are caused by acute thoracic empyema or tuberculous pleuritis. Open thoracotomy and decortication are traditional treatments for chronic empyema. However, some cases, such as those with thick calcifications around a large cavity, may be difficult to decorticate in a single surgery. We successfully treated a case of chronic empyema with a large cavity surrounded by a thick calcified membrane that was peeled off gradually each day through fenestration of the thoracic cavity with negative-pressure wound therapy (NPWT).

**Case presentation:**

The patient was a 47-year-old man who had undergone thoracic drainage for left post-pneumonia empyema at another hospital 10 years previously. He presented to our hospital with a fever of 39 °C, bloody sputum, and severe fatigue for 3 days. Computed tomography showed a 9-cm mass shadow in the left intralobar space and an adjacent 21 × 15 × 9-cm fluid-filled calcified unilocular cavity up to 5 mm in thickness. He underwent thoracic drainage for fluid, and empyema was suspected; the fluid was foul-smelling and purulent. The patient did not improve with antibiotics and intrathoracic lavage; therefore, thoracoscopic decortication was performed. The thoracic cavity had a thick calcified membrane filled with dark-red slurry resembling old blood. We attempted decortication; however, the calcified membrane was difficult to remove. Two drains were used for the pleural lavage. However, no improvement was observed with intrathoracic lavage and drainage; therefore, a fenestration was performed. The calcified membrane was peeled off each day for 3 months. Gradually, granulation increased and the inflammatory reaction improved. After NPWT, the empyema cavity gradually shrank to 8 cm × 6 cm × 2 cm. A latissimus dorsi flap closure was performed, and the patient was discharged.

**Conclusions:**

This is an informative report on the daily decortication of a highly calcified purulent membrane using NPWT in a patient with chronic empyema. The description of this method will aid in the management of patients with chronic empyema and thick calcified membranes.

## Background

Chronic empyema (CE) is characterized by thickened visceral and parietal peels that hamper the ability of the affected lung to re-expand. It requires definitive surgical intervention, decortication with or without lung resection, and/or pleural space obliteration procedures such as thoracoplasty and the use of various muscle flaps [1, 2]. Currently, open thoracotomy and lung decortication are the most favored approaches [3]. However, in chronic calcified pleural empyema, decortication remains challenging or even impossible because of the presence of thick calcified pachypleuritis and the risk of high-volume blood loss [4]. We describe a case of successful treatment of CE with a large cavity surrounded by a thick calcified purulent membrane (CPM) of the thoracic cavity that peeled off gradually each day through fenestration with NPWT.

## Case presentation

**Fig. 1:**
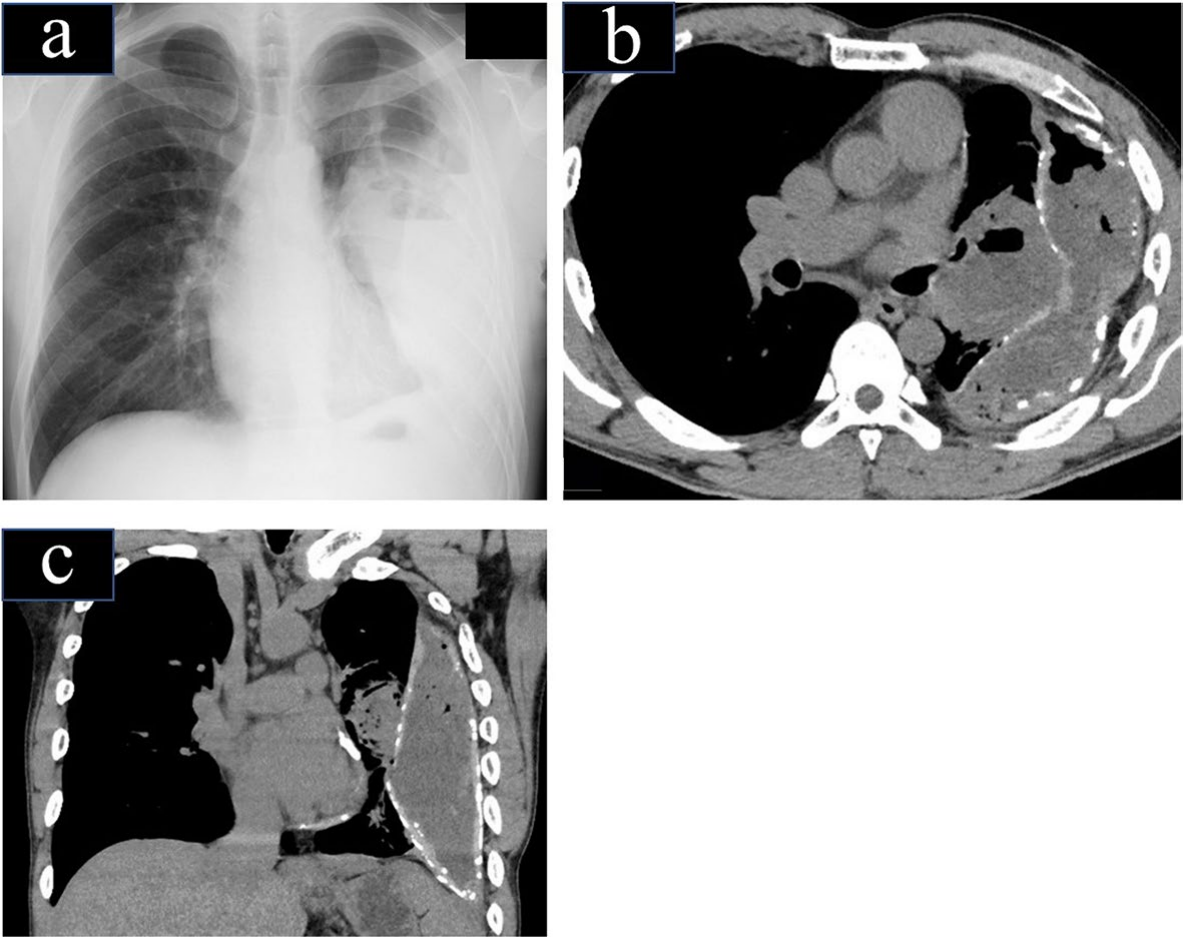
Imaging findings on admission. **a** Chest X-ray findings on admission showed pleural effusion from the left middle lung field to the lower lung field, including air–fluid levels. **b** Chest CT (horizontal section) revealed an extrapulmonary lesion with fluid and gas inside, accompanied by calcification in the surrounding area. **c** In a coronal section, extrapulmonary lesions were observed from the second rib to the diaphragm

A 47-year-old man with CE was admitted to our hospital with a fever of 39 °C, bloody sputum, and severe fatigue for 3 days. He had undergone left thoracic drainage for acute empyema after pneumonia at another hospital 10 years prior. He was in poor general condition with edema in both feet and jaundice. Oxygen saturation was 93% in room air. Laboratory findings on admission are presented in Table 1. Chest radiography on admission showed a large amount of left pleural effusion with air–fluid levels (Fig. 1a). A mass-like lesion 9 cm in size was detected in the left intralobar space on chest computed tomography (CT) (Fig. 1b, c). A bulky extrapulmonary lesion (21 × 15 × 9 cm) filled with a gas-containing fluid was also detected. A large extrapulmonary unilocular space surrounded by thick CPM was observed. Thoracic drainage was performed, and purulent fluid was drained. The patient was diagnosed with acute exacerbation of CE and hospitalized. *Actinomyces odontolyticus* and *Fusobacterium necrophorum* were detected in the purulent fluid. Antibiotic therapy with sulbactam/ampicillin was then initiated. After 1 week of treatment, the fever and inflammatory reaction did not improve, and the drained fluid remained purulent. Based on our discussion with the respiratory physicians, surgery was performed because the patient’s general condition had slightly improved, and conservative treatment was difficult.

**Table 1:** GTCS cases

WBC	19200	μ/L	BUN	62	mg/dL
RBC	464x10^4^	μ/L	AST	19	IU/L
Hb	14.2	g/dL	ALT	19	IU/L
Plt	24.1x10^4^	μ/L	Na	126	mEq/L
TP	6.9	g/dL	K	3.3	mEq/L
Alb	3.1	g/dL	Cl	87	mEq/L
Cre	2.03	mg/dL	HbA1c	5.6	%

### Operative findings of the 1st surgery

**Fig. 2:**
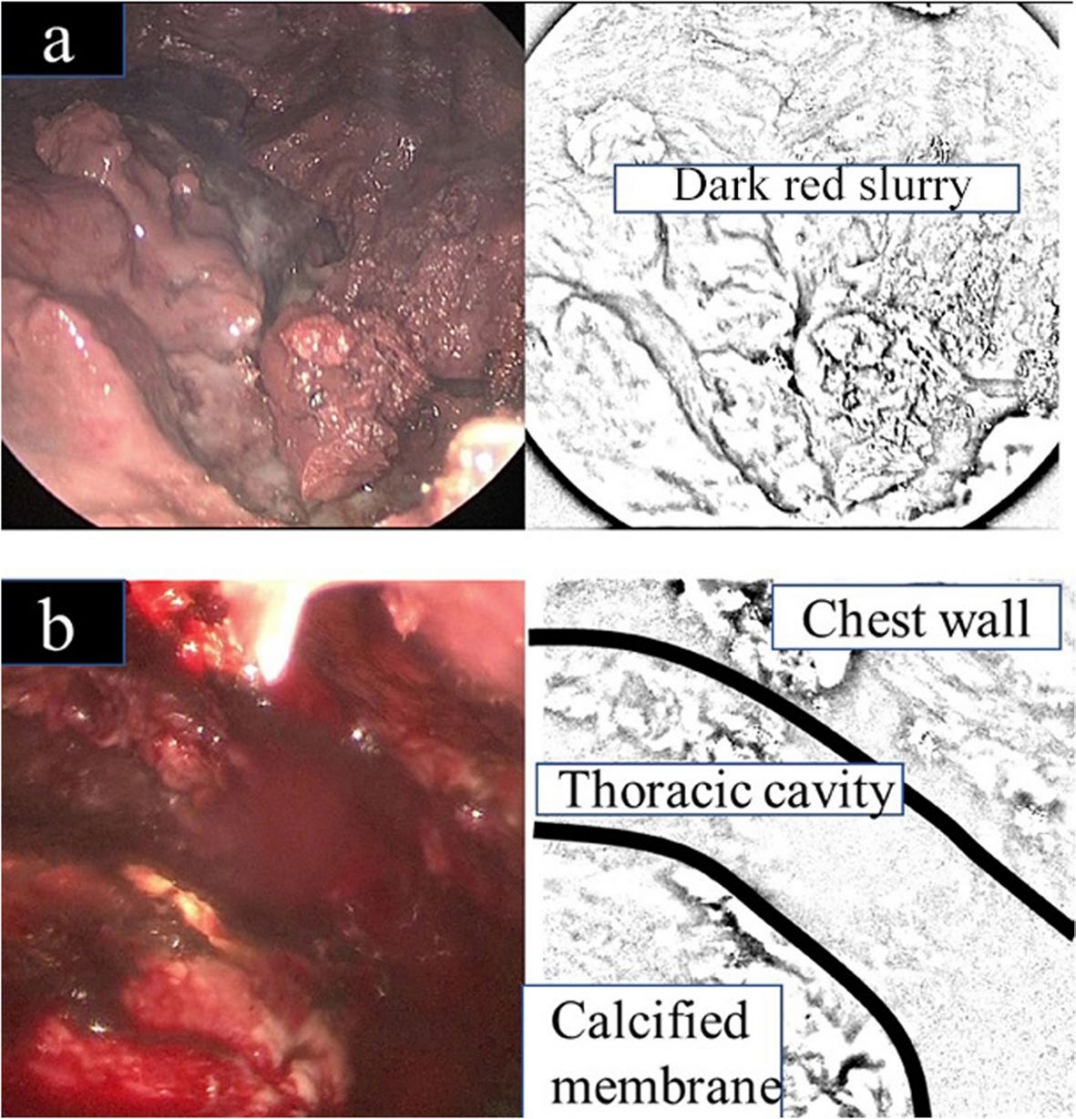
Intraoperative findings of first surgery. **a** A dark-red slurry, suggesting old bleeding, was found in the empyema cavity. **b** After removing the dark-red slurry, the visceral pleura and chest wall were covered with a thick, calcified purulent membrane

Under general anesthesia, we opened the 5th intercostal space with a 10-cm skin incision. The thoracic cavity had a thick CPM and was filled with a dark red slurry resembling the old blood. We attempted to perform decortication, but the CPM was difficult to remove (Fig. 2a, b). We searched for an interlobar mass-like lesion during surgery; however, the location of the lesion could not be identified. During the surgery, no obvious air leaks were observed. After irrigation of the intrathoracic space, two drains (a 32-Fr silicon tube drain and a 24-Fr BLAKE® Drain; Ethicon, Tokyo, Japan) were placed and the surgery was completed.

### After the 1st surgery

**Fig. 3:**
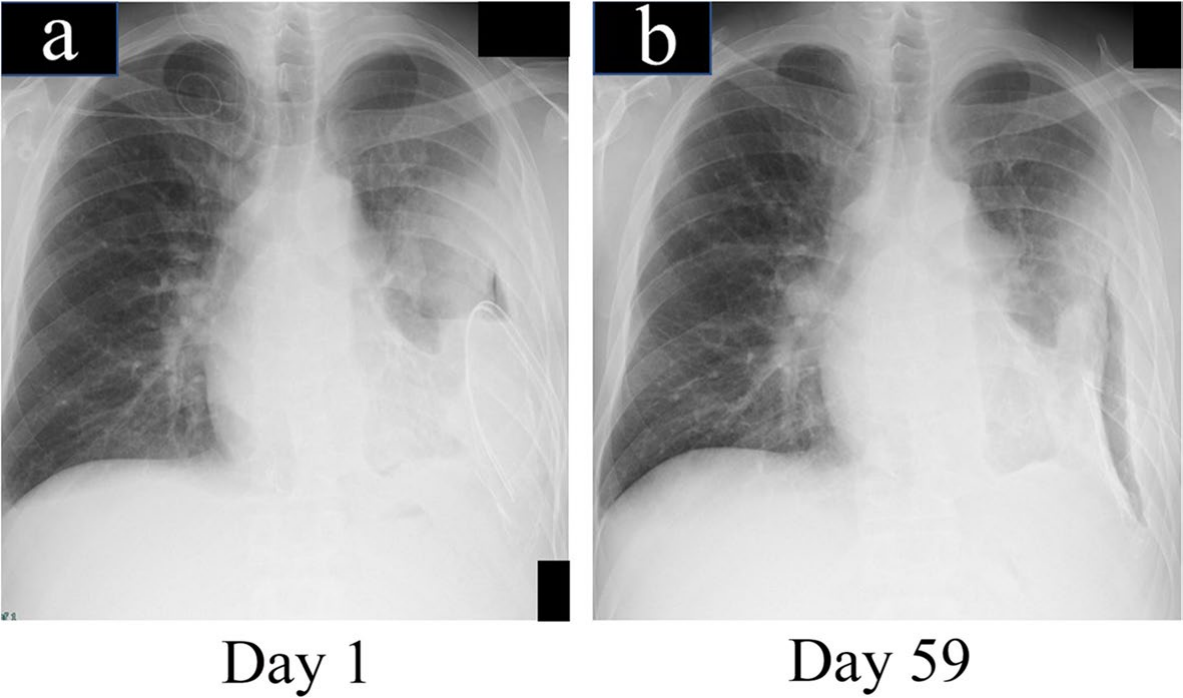
Postoperative X-ray findings after the first surgery. **a** Chest X-ray on the first day after the first surgery. **b** Chest X-ray with negative-pressure wound therapy, 59 days after the 1st surgery

From the day after the first surgery, intrathoracic lavage was performed using 1000 ml of sterile water twice daily for 39 consecutive days. Treatment with a negative-pressure maintenance device (Renasys®; Smith and Nephew, Tokyo, Japan) was started at − 140 mmHg to apply strong suction pressure through the drain into the thoracic cavity, thereby reducing the cavity while taking care to avoid exacerbating the infection on postoperative day 41. However, despite the strong suction pressure, no significant reduction was observed in the empyema cavity (Fig. 3a, b). Fenestration was performed 60 days after the initial surgery.

### Operative findings of the 2nd surgery

**Fig. 4:**
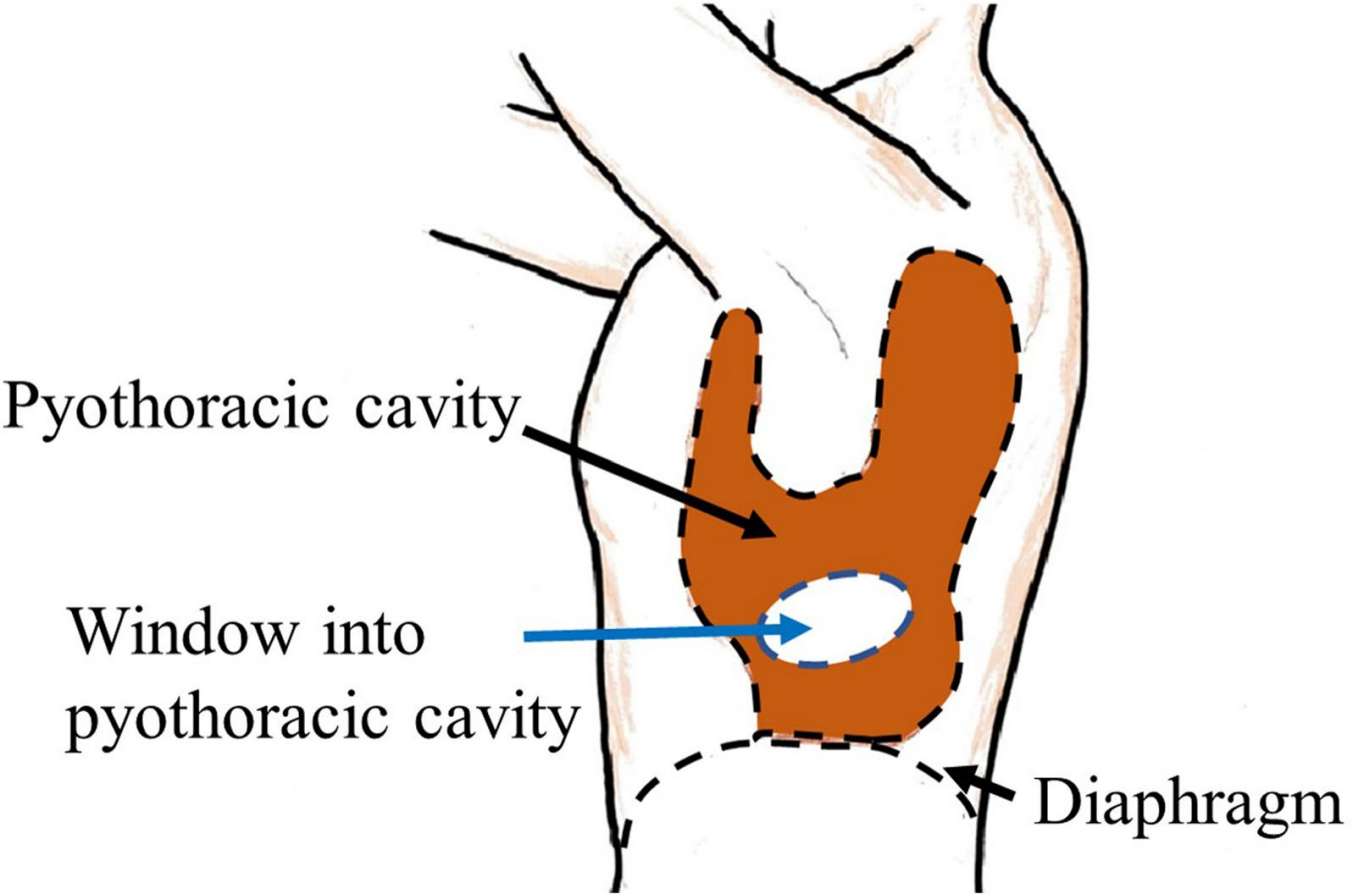
Intraoperative findings of second surgery (fenestration surgery). Part of the left upper lobe was adhered to the chest wall on the cranial side, and the empyema cavity was divided into anterior and posterior parts

The second surgery was performed under general anesthesia. A 15-cm skin incision was made in the left 6th intercostal space, and parts of the 6th, 7th, and 8th ribs were excised. The empyema cavity was divided into anterior and posterior parts (Fig. 4). Finally, 17 pieces of gauze were inserted for drainage and the fenestration was completed.

### After the 2nd surgery

**Fig. 5:**
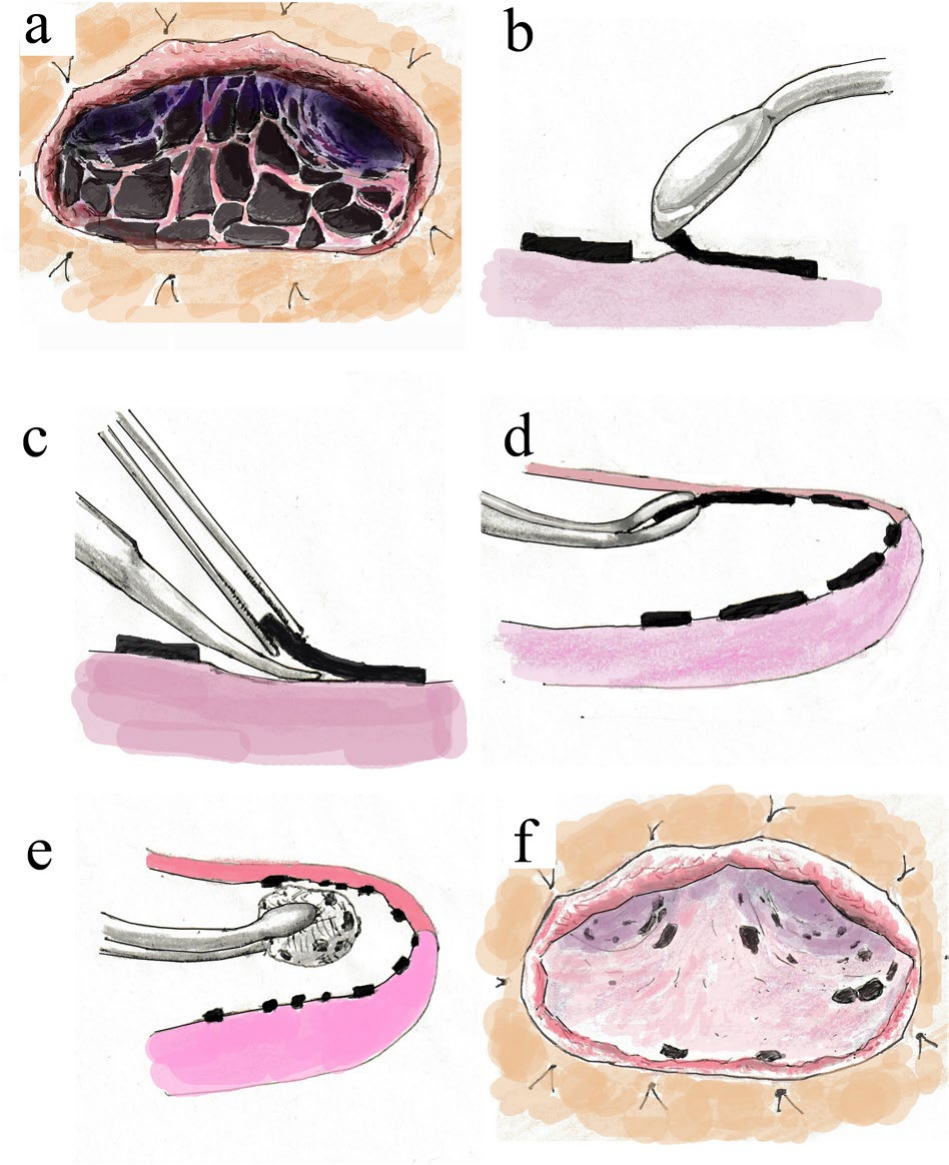
How to remove the calcified purulent membrane. As the cavity became cleaner, the CPM became brittle and cracked (**a**) When we rubbed it with a blunt spoon, the edge of the CPM became stuck. We then grasped the edge of the CPM with forceps and peeled it off with Cooper scissors (**b**, **c**). The CPM that came off deep inside the cavity was removed by grasping it with the sponge forceps (**d**). We used a sponge forceps with long curved tips to hold gauze in hard-to-see areas on the apex of the lung and apical wall of the thoracic cavity and scraped the chest wall and lung surface to remove the CPM that had come off (**e**). After 77 days of decortication, 80% of the CPM was removed (**f**)

**Fig. 6:**
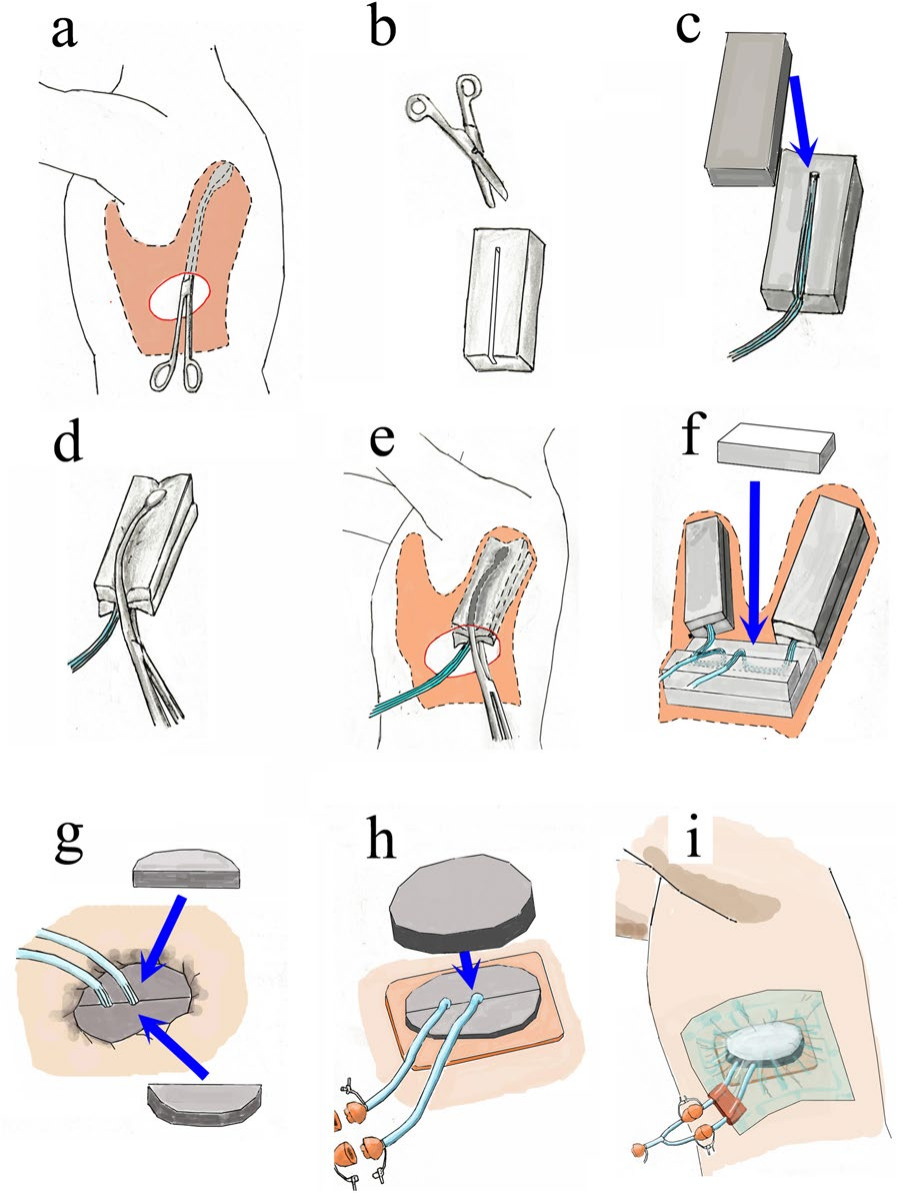
The use of NPWT in the present case. The depth of the cavity was measured with a sponge forceps (**a**). Foam filler was cut to an appropriate size and a groove was made in the center of the filler using Cooper scissors (**b**). A 15 Fr channel drain was placed in the groove of the foam filler, and another foam filler was placed on the drain to sandwich it (**c**). The foam filler was grasped with sponge forceps and placed inside the cavity (**d**, **e**). Two drains were placed, one in the anterior cavity and the other in the posterior cavity, with an appropriately sized foam filler placed at the bottom of the proximal cavity, and a half-sized foam filler placed on top of it on the caudal side. The drain was lined up at the exit of the wound as shown. A half-sized foam filler was placed on the cranial side and the drain was sandwiched between the foam fillers to prevent shifting (**f**). The filler cut into a semicircle is placed so as to sandwich the drain (**g**). To protect the wound edges, the wound is covered with Hydro site® (Smith and Nephew, Tokyo, Japan) and covered with filler cut into a circular shape so that the drain groove does not protrude outside the filler (**h**). Finally, the wound is covered with a drape, the two drains are connected to the Y connector, and suctioning is initiated (**i**)

**Fig. 7:**
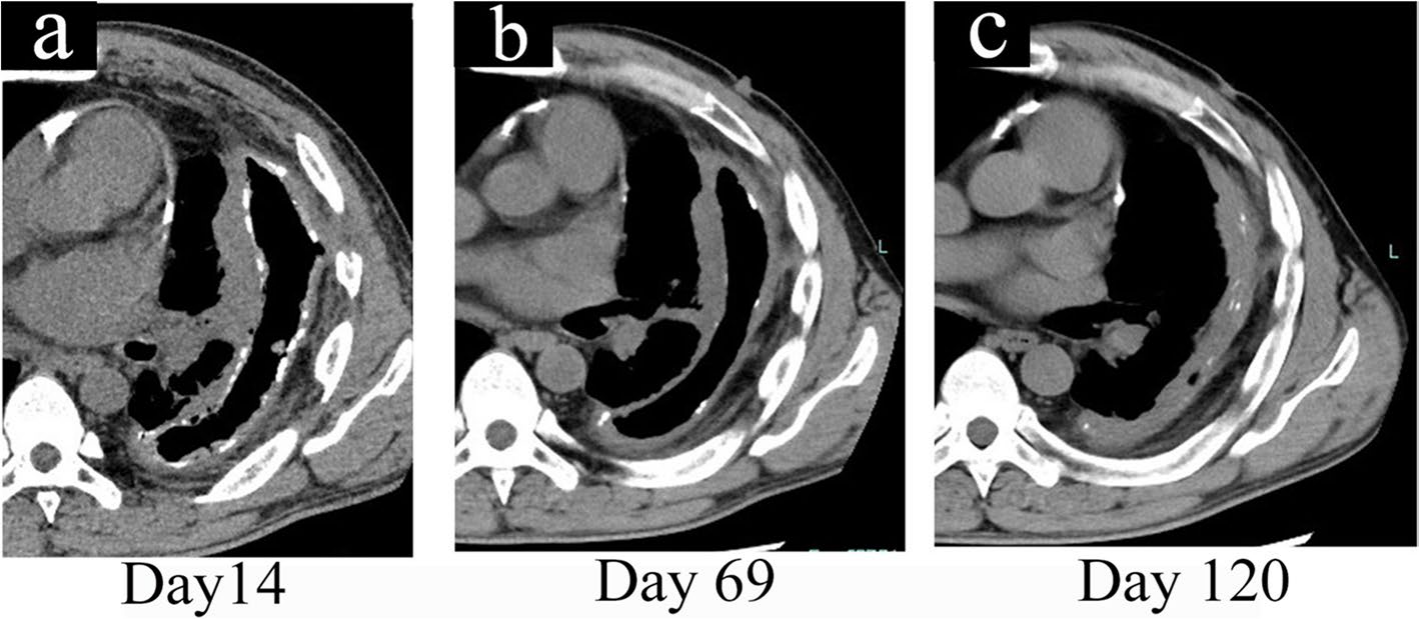
Postoperative CT findings of the second surgery (fenestration surgery). Postoperative CT showed that there was a reduction in the calcified pleura and empyema cavity with lung expansion by daily decortication for 77 days followed by NPWT for 43 days. **a** 14 days after the 2nd surgery. **b** 69 days after the 2nd surgery. **c** 120 days after the 2nd surgery

After the second surgery, we peeled the CPM slightly and changed the gauze for drainage through the fenestration site twice daily for 77 days. As the cavity became cleaner, the CPM became brittle, cracked, and easily peeled off. Details of the CPM removal method are shown in Fig. 5. One tip to avoiding lung injury and bleeding is to leave areas that are resistant to decortication. After 77 days of decortication, 80% of the CPM was removed (Fig. 5f). After the culture result became negative, NPWT was restarted using Renasys® at − 140 mmHg. Details of the use of NPWT in this case are shown in Fig. 6. The device was replaced twice a week. In addition, we removed the residual CPM during the foam filler replacement. After 43 days of NPWT, the cavity shrunk significantly (from 21 × 15 × 9 cm to 8 × 6 × 2 cm; Fig. 7). On the 128th day after the 2nd surgery, latissimus dorsi flap closure was performed.

### After the 3rd surgery

**Fig. 8:**
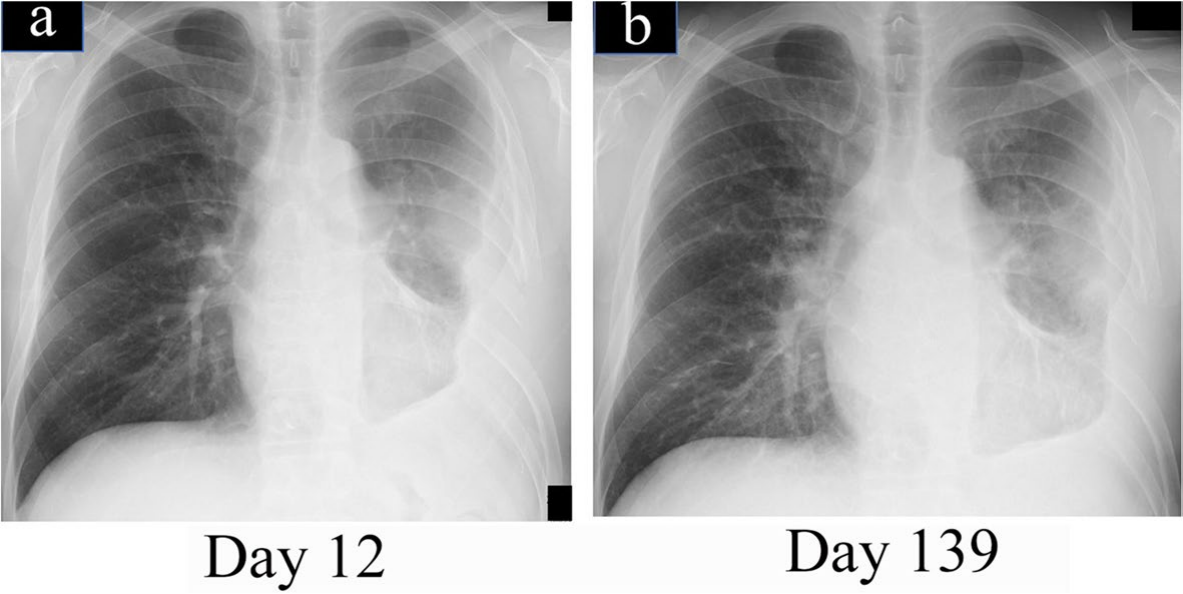
Changes in chest X-ray after closure surgery. Chest X-ray at discharge (12 days after closure surgery). **b** 4 months after discharge (139 days after closure surgery). Improvement in pulmonary expansion was observed

The patient’s course after thoracoplasty was good, and the was discharged 12 days after the third surgery (day 209 of admission). Chest radiography performed as an outpatient showed further improvement in his left lung expansion and thoracic empyema with no recurrence 3 years after discharge (Fig. 8).

## Discussion

Pleural empyema is divided into three stages: exudative, fibrinopurulent, and organizing [5]. Treatment included thoracic tube drainage, antibiotic administration, and respiratory physiotherapy. In the first two stages, surgery can be avoided. However, surgery is often required during the organizational stage. The disease is called chronic empyema (CE) when more than 3 months have passed since its onset. If proper treatment is not provided until the chronic stage, the condition progresses to the development of a calcified pleural pocket with intercostal retraction and a pleurocutaneous fistula. Management becomes more difficult and time-consuming for both the patient and surgeon [6]. The underlying mechanism is assumed to involve old pleuritic lesions that exist as dead spaces and secondary infections in the later years. The main symptoms were fever, compression, and fistula [7]. In this case, the patient had undergone treatment for empyema approximately 10 years previously. We speculated that a dead space was present after the treatment, allowing secondary infection to develop in the cavity. Thickened pleura, collapsed chest wall, and accumulation of purulent fluid in the thoracic cavity are typical findings [8]. Two of the three typical findings were noted in this case (the exception was a collapsed chest wall). This case resembles chronic expanding hemangioma in the point that the patient had bloody sputum and lesions with a capsule. However, chronic expanding hemangioma has a rich blood flow and embolization of the feeding blood vessels is required before surgical removal [9]. When we drained the patient's chest cavity the drainage fluid was purulent, not bloody. Furthermore, there was no bleeding at all when we removed the calcified purulent membrane (CPM) after fenestration. In these two points, we considered this case to be different from chronic expanding hemangioma. Harmouchi et al. reported the benefits of surgery in young patients with symptomatic chronic and calcified pleural empyema, especially in the presence of a bronchopleural fistula. Decortication is generally considered a basic surgical intervention for CE. It releases the underlying lung by removing thick pachypleuritis to correct the patient’s ventilation [6]. In this case, it was difficult to remove the thick CPM during initial surgery. In acute empyema, even if some cavities remain, the empyema will improve if the thoracic cavity can be sterilized. Although the initial diagnosis was CE, we expected a therapeutic effect similar to that of acute empyema with intrathoracic irrigation and strong suction pressure therapy after decortication, to reduce and sterilize the thoracic cavity. CPM not only facilitated the re-expansion of the lung but also prevented granulation growth. This makes it impossible to reduce cavities. Fenestration was performed and the empyema cavity became cleaner with daily peeling and gauze drainage. Thoracoplasty was performed after cavity reduction, and the patient was discharged. There are a few reports of treatment up to the closure of empyema with CPM, and the present case represents our first experience with this course of treatment. The American Association for Thoracic Surgery (AATS) expert consensus guidelines weakly suggest that vacuum-assisted closure (VAC) therapy be used in patients with chronic empyema as an alternative to debridement with daily gauze changes after fenestration [10]. However, it states that caution should be taken in patients with tracheal pleural fistulas or pulmonary fistulas, as this may increase air leakage. Nishii et al. reported that 10 cases of empyema with bronchopulmonary fistula could be treated with NPWT after closure with suture closure using muscle flaps or bronchial occlusion [11]. In Japan, we can only make a medical insurance claim when we use the material for local negative pressure closure treatment as a filler during NPWT. This local negative pressure closure treatment material is normally available for up to 3 weeks, and can be used for up to 4 weeks if necessary for medical reasons (http://www.jacsurg.gr.jp/committee/guideline_em.pdf). NPWT was highly effective when reapplied after most CPM had been removed. NPWT led to lung re-expansion and adhesion between the lung and the chest wall in the cavity. This also promotes granulation growth, leading to a reduction in the cavity. The first case of intrathoracic NPWT was reported in 2006. Varker et al. successfully managed a patient with post-lobectomy empyema using a VAC device after open debridement of the empyema cavity [12]. However, their case lacked a CPM and the situation is different from ours. No obvious air leak was observed from admission to discharge because the device activates an alarm when it detects an air leak, but the alarm was not activated in our case. In our case, NPWT was considered to be a very effective method that led to successful wound closure.

## Conclusions

In the curative treatment of a patient with CE and large cavities surrounded by CPM, peeling each day after fenestration and NPWT was effective.

## Data Availability

All data generated or analyzed in this study are included in this manuscript.

## Abbreviations

CE: Chronic empyema
CPM: Calcified purulent membrane
CT: Computed tomography
NPWT: Negative-pressure wound therapy
VAC: Vacuum-assisted closure
